# Non-Conventional Wing Structure Design with Lattice Infilled through Design for Additive Manufacturing

**DOI:** 10.3390/ma17071470

**Published:** 2024-03-23

**Authors:** Numan Khan, Valerio Acanfora, Aniello Riccio

**Affiliations:** Department of Engineering, University of Campania “Luigi Vanvitelli”, via Roma, 29, 81031 Aversa, Italy; valerio.acanfora@unicampania.it

**Keywords:** additive manufacturing, lattice-infilled design, lightweight structures, non-conventional wing structure, finite element analysis

## Abstract

Lightweight structures with a high stiffness-to-weight ratio always play a significant role in weight reduction in the aerospace sector. The exploration of non-conventional structures for aerospace applications has been a point of interest over the past few decades. The adaptation of lattice structure and additive manufacturing in the design can lead to improvement in mechanical properties and significant weight reduction. The practicality of the non-conventional wing structure with lattices infilled as a replacement for the conventional spar–ribs wing is determined through finite element analysis. The optimal lattice-infilled wing structures are obtained via an automated iterative method using the commercial implicit modeling tool nTop and an ANSYS workbench. Among five different types of optimized lattice-infilled structures, the Kelvin lattice structure is considered the best choice for current applications, with comparatively minimal wing-tip deflection, weight, and stress. Furthermore, the stress distribution dependency on the lattice-unit cell type and arrangement is also established. Conclusively, the lattice-infilled structures have shown an alternative innovative design approach for lightweight wing structures.

## 1. Introduction

The aerospace industry places significant importance on lightweight structures with a high stiffness-to-weight ratio. However, conventional manufacturing methods often result in over-designed parts, leading to unnecessary weight [1]. Fortunately, today’s additive manufacturing (AM) presents opportunities for manufacturing structural designs that were previously impossible, including intricate lattice structures [2]. AM is extensively utilized across many industries, including aerospace, automotive, and medical sectors, owing to its numerous advantages such as accelerated product development cycles and the ability to produce components with intricate geometries with reduced cost and lead times [3]. Among many components of aircraft, the wing structure is always considered the most important part because of the high-stress demand. Wings attached at one end to the fuselage of the aircraft and free on the other side, acting as cantilever beams, are the primary load-carrying structures. They are subject to different types of loads such as lift, fuel, the engine, landing gears, and inertial, structural, and other aerodynamic loads [4].

Wing structural optimization is a challenging task involving many individuals and its own design parameters and constraints. There has been significant research reported on reducing the weight and increasing the aeroelastic performance of the aircraft wing [5,6,7,8]. The structural optimization of aircraft wings using numerical optimization has been traced back [9,10,11,12]. There are different ways of conducting wing structural design. One of the most common, conventional ways is arranging the wing box components’ spars and ribs in a perpendicular outline. The proper adjustment of the location and thickness of these components can lead to a lightweight structure with improved mechanical performance. However, this method is limited to keeping the location, orientation, and number of components fixed during the entire optimization process [13,14,15,16,17]. Another method is to keep the thickness constant and adjust the position and orientation of the wing box components, leading to an optimized structure with curved structures that can be treated as both spar and ribs [18,19]. Despite satisfactory design from this method, there is a possibility of ignoring possibly more efficient designs. Similarly, the other limitation of this method is the assumption that the FE model used depends on shell-based elements, which assumes that the wing box is composed of shell-based plates [1].

Recently, topology optimization (TO) has been extensively used for wing structure optimization by incorporating input from additive manufacturing technologies [19,20,21,22,23]. The TO design method basically updates and locates the structure weight based on the design variables at each step of the iteration through an optimization formulation [20]. The resulting structures achieved through optimization techniques often exhibit similarities to natural designs [19], differing from traditional rib and spar configurations. However, it is important to note that this does not reduce the value of the traditional thin-walled spar–ribs configuration. On the other hand, with proper application of loading conditions and other restraints, there is a possibility that the topology optimization may result in structures closely resembling the conventional wing box structure [1]. This conventional wing box design might not be able to meet the increasingly demanding criteria for both strength and weight. Consequently, this design method could possibly confine the maximum performance proficiencies of the airplane. Alternatively, it is also believable that the conventional wing box structure enforces design and manufacturing limitations that should be exceeded for improved structural design considerations [1].

Due to these limitations, designers are exploring non-conventional structural concepts driven by the utilization of advanced optimization methods such as lattice-infill structures and additive manufacturing techniques [24,25,26]. These methodologies present the flexibility to purposefully decide the optimum arrangement of materials within the design area of the wing [27]. By taking advantage of these advancements, designers can make necessary decisions regarding the most effective use of materials in the wing’s design. On the other hand, high-performance lattice structures with lightweight and extraordinary capabilities often acquire intricate geometries, which can limit their feasibility using conventional manufacturing methods. However, additive manufacturing has proven its convincing potential to bring designers closer to achieving the desired performance of such structures [28,29,30]. In contrast to conventional manufacturing approaches, AM techniques offer enormous potential for producing components with intricate geometries [31,32]. This astonishing flexibility makes AM a favorable technology for generative design in the development of customized, multi-scale, and multi-material high-performance structures [33]. While the manufacturability of unusual, complicated structures is no longer a critical concern when employing AM, the development of new opportunities calls for more refined and efficient design approaches. For this purpose, considerable research has focused on computational optimization for the design of high-performance lattice structures in the literature [34,35,36,37,38,39,40,41].

For example, lattice structures have valuable applications as load-bearing elements, including fuselage rings, wing and stabilizer ribs, spars, cabin floor beams, and more [42]. Specifically, lattice shear webs employed in wings and stabilizers exhibit excellent weight efficiency, being notably lighter compared to aluminum prototypes [43]. Likewise, a study conducted by [44] achieved significant weight reduction in aircraft structures by using lattice structures in the stabilized spar, which can be also used for wing transverse ribs and fuselage floor beams. Moreover, Seung et al. [25] investigated the identification of an optimum truss lattice design for deployable UAV wings. The anticipated three different types of lattice designs were fabricated using a 3D printer utilizing a polypropylene-like photopolymer. The compression testing of inflatable wing designs reveals the incorporation of compliant mechanisms and deployable structures, leading to maximum flexibility in the design of UAV wings. Their study suggested future investigation and numerical simulation for exploring further structural properties including tensile and shear strengths. Furthermore, lattice structures have been successfully implemented in many aerospace parts [45]. For example, Fasel et al. [46] showed the successful use of additive manufacturing in manufacturing and flight-testing morphing aerospace structures, emphasizing the potential of AM to improve lightweight design and flight performance. Opge-noord and Willcox [47] anticipated a lightweight design method for aircraft brackets using additively manufactured cellular structures, employing topology optimization and selective laser melting (SLM) manufacturing techniques. Research by Spadoni, et al. [48] explored a novel chiral-based lattice structure for aircraft wings, demonstrating its ability to endure significant deformations without surpassing yield strain. Magna Parva [49] introduced a truss-based 3D lattice structure for landing buffer systems in re-entry capsules, elaborating its capacity in aerospace applications. The method features the realization of adaptive structures and mechanisms, enabling increased elastic and global shape deformation in response to external loading. Similarly, another innovative technique for actively deformable wings was presented, offering the advantages of low-density, spatially tuned, stiff, and highly compliant structures. Furthermore, the span-wise twist deformation with lightweight enhanced aerodynamic performance and easy production of the morphing wings were reported through a case study [50]. The planer lattice structures are adopted in the skin of the morphing wing of an airplane using the homogenization technique to optimize the performance of the lattice to obtain enhanced mechanical properties under applied loading. They highlighted that the concept of a morphing wing must undergo significant deformations while maintaining shape and strength during morphing, particularly in cord and curvature changes [51]. The effect of lattice orientation on the mechanical properties of lattice-infilled morphing skin is reported. Analyzing five different types of lattices, the honeycomb lattice recorded better performance in the camber direction, while the square lattices presented better in-plane sheer performance [52].

Despite the many applications of lattice-infill design in airplane skin panels and morphing wing structures, there is limited work, to the knowledge of the authors, on the utilization of infilled lattices for wing structures—especially for the replacement of conventional wing boxes with non-conventional lattice-infill structures. Therefore, this study demonstrated the structural performance of beam-based lattices infilled for a small Unmanned Aerial Vehicle (UAV) wing structure as a case study. UAVs have undergone significant advancements and found application in diverse military and civilian sectors. These applications encompass reconnaissance, combat missions, surveillance of critical infrastructure, exploration of celestial bodies, and disaster management. In this study, five different lattice structures are used as an infill of the wing structure, completely replacing the conventional wing box structures. The parametric optimization of lattice structure size is performed with unit cell type and arrangement as design variables. The commercial software tool nTop (nTopology) [53] is used for the development and optimization of lattice design, followed by the structural performance simulation of optimized structures in the ANSYS workbench.

A detailed description of wing structure, material properties, lattice configuration framework, and numerical simulation are presented in Section 2; followed by the numerical results and discussion in Section 3; finally, the conclusion is provided in Section 4 of the paper.

## 2. Materials and Methods

**Figure 1:**
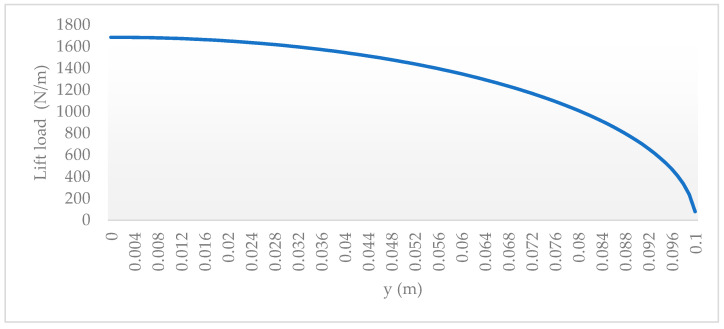
Lift distribution over the half-span wing calculated using the Schrenk method.

The numerical simulation of wing structures is performed on an ANSYS workbench 21. Initially, the base wing modeled with the conventional spar–rib was analyzed for wing-tip deflection and to determine if von Mises stress was less than the allowable design stress (167 MPa). This allowable stress is calculated from the tensile yield strength (Table 1) of the material used with the factor of safety 1.5. The general characteristic of the UAV considered is given in Table 1, while the final detail dimensions of the base-wing design are presented in Section 2.1. The corresponding variable wing lift load was obtained using Schrenk’s Approximation [54,55,56] for design optimization, as shown in Figure 1. The lift distribution is calculated at level flight conditions as per the European Aviation Safety Agency (EASA) CS–VLA [57] recommendation with the load factor of possible extreme conditions [58,59]. The optimized lattice-infilled structures were also analyzed for tip deflection, stress distribution, and concentration.

### 2.1. The Structural Geometry of the Wing

**Figure 2:**
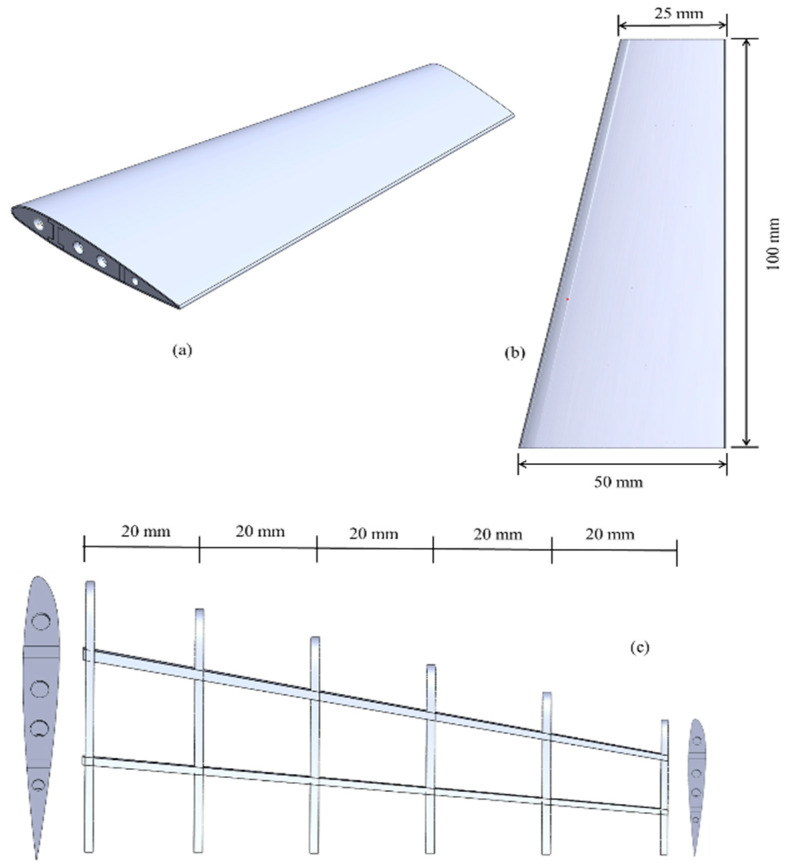
Base-wing structure (**a**) Isometric view of the entire wing, (**b**) Dimensions of the wing with skin, (**c**) Internal spar–ribs frame with starting and end profile.

The wing structure under consideration as a case study represents a small UAV with a 200 mm wingspan designed for easy metal additive manufacturing. These compact UAVs, often referred to as drones, have attracted substantial interest in both military and civilian domains due to their recognized advantages of improved stability and extended operational endurance [60]. The conventional base-wing structure consists of two spars, six ribs, and a skin. For skin and ribs airfoil, the NACA 23012 profile was used [61]. The thickness of the leading spar was kept to 3 mm, while that of the rear was 2 mm. The thickness of equally spaced ribs was considered to be 1.5 mm, while the skin thickness was kept constant at 0.4 mm. The detailed dimension of each component is given in Figure 2. The total wing weight reported from the CAD geometry was 19 gm.

### 2.2. Material

The material considered in this analysis is AlSi10Mg. Advanced aluminum alloys are preferred for lightweight aerospace components due to their high strength, ductility, corrosion resistance, cost-efficiency, and ease of manufacturing [62,63]. Heat treatment advances enable these alloys to compete effectively with advanced composites in various aerospace applications [64]. The recent development in metal additive manufacturing made possible the easy manufacturing of complex lattice structures with such alloys, and they have been extensively used in aerospace components [65,66]. The alloy’s composition, featuring Si and Mg elements, enhances powder fluidity, mitigates issues like hot cracking, and contributes to aging strengthening, making it a preferred material for the AM of lattice structures [67,68,69,70]. This has been used by many researchers for manufacturing lattice structures using metal additive manufacturing [71,72,73]. The material properties used in the numerical simulation are presented in Table 2 [74,75].

### 2.3. Lattice Configuration and Optimization Framework

The mechanical properties of lattice structures can be altered by varying numerous aspects, such as unit cell topology, sizes, and arrangement. These parameters play a significant role in predicting the mechanical behavior of lattice structures. Furthermore, by fixing the base material and relative density, these properties primarily depend on the architecture of the unit cell, aiming to maximize the stiffness/strength-to-weight ratio [76]. Various studies [77,78,79] have emphasized the significance of unit cell arrangement enhancing mechanical properties, focusing on achieving optimal mechanical properties with minimal weight. Therefore, the goal is to derive an optimal lattice structure, consisting of optimal unit cell arrangement under bending loading conditions, as selecting the appropriate lattice topology poses a significant challenge. The customization capabilities offered by additive manufacturing exceed the capabilities of many commercially available CAD softwares. Conventional CAD design software is well-matched for conventional manufacturing approaches and often lacks the complexity required for AM [80]. To overcome this limitation, a software called nTop (commonly known as nTopology), developed in New York, USA [53], was utilized. nTop employs implicit modeling techniques to design complex lattice structures proficiently, offering faster creation and requiring less space compared to software utilizing explicit modeling techniques. With nTop, lattice structures can be generated in various arrangements, including surface-based and beam-based designs in cartesian, cylindrical, and spherical configurations. The employment of implicit modeling and unified finite element analysis (FEA) within nTop software pointedly streamlines the process of generating design-driven lattice structures compared to other CAD packages. The software’s integration with MATLAB or Python coding permits greater control over the design output, simplifying customization and personalized design solutions. 

**Figure 3:**
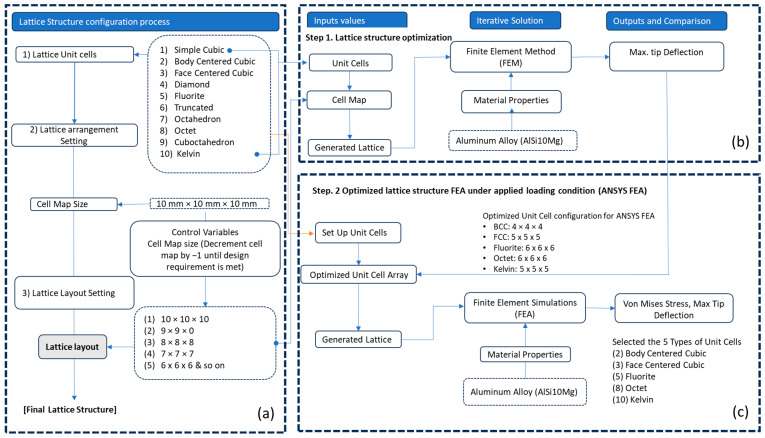
Lattice configuration optimization method and general study framework, (**a**) Lattice generation and configuration, (**b**) Arrangement optimization, (**c**) FEA simulation.

**Figure 4:**
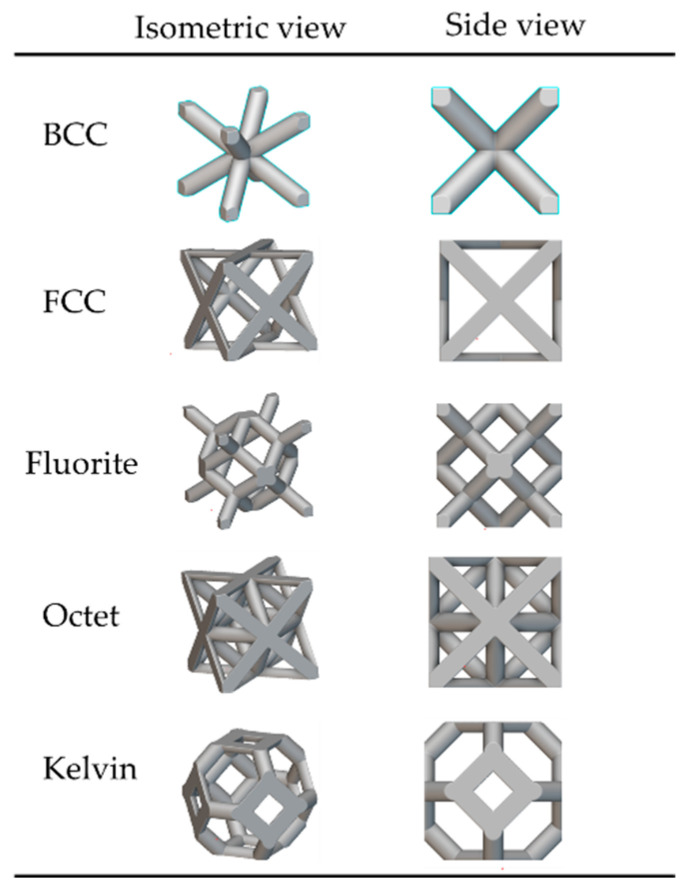
Unit cell topology.

**Figure 5:**
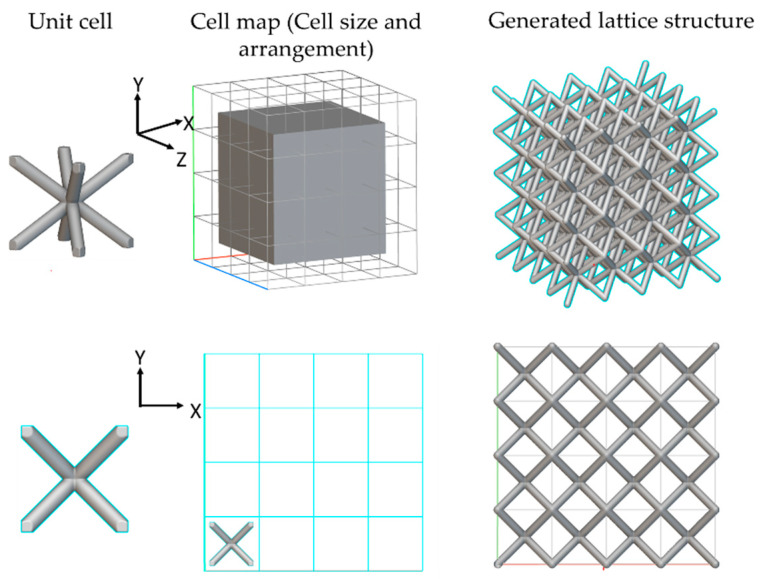
Lattice structure generation process.

**Figure 6:**
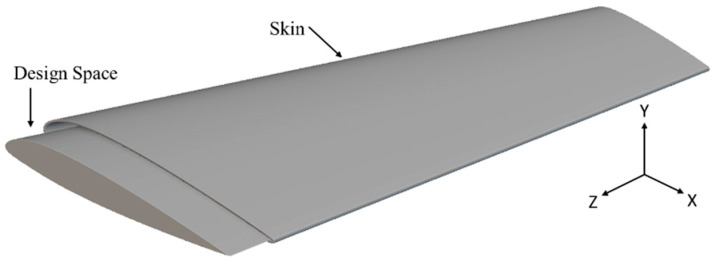
Lattice wing design space with skin.

To overcome the optimal unit cell type and arrangement challenge under design loading, a general arrangement optimization and analysis framework is described in Figure 3. As mentioned in Figure 3, the framework utilizes the generative design capabilities of nTop to perform cell arrangement in the design space for different unit cell topologies, as mentioned in Figure 4. This arrangement optimization is controlled by the cell map defining the unit cell size, as elaborated in Figure 5. Starting from the initial cell size of 10 × 10 × 10 mm for x, y, and z directions respectively, the iterative simulation is performed (Figure 3a) for each unit cell while checking the design criteria of base-wing max tip deflection (d < 1.6 mm). The unit cell size is decremented by 1 mm for each iteration and continues until the tip deflection value in the generated lattice arrangement is under the base model value (Figure 3b). This leads to the optimum lattice arrangement for the applied load, with minimum weight and tip deflection compared to the base model. To automate the iteration, a Python code was developed and utilized to perform this iterative unit cell arraignment fulfilling the tip deflection criteria. Furthermore, the optimum lattice arrangement is simulated on the ANSYS workbench 21 under the same applied load for stress distribution and concentration (Figure 3c). The wing design for the lattice infill to be converted into an optimal lattice structure by different unit cells is shown in Figure 6.

**Figure 7:**
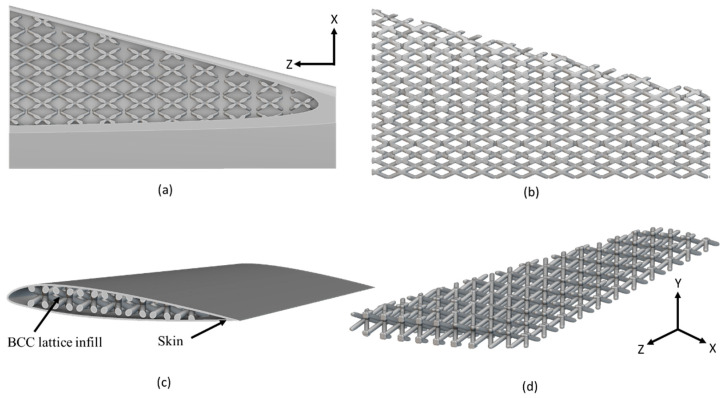
4 × 4 × 4 mm BCC lattice-infilled wing, (**a**,**b**) represents the plain view, (**c**,**d**) showing the isometric view with and without skin.

**Figure 8:**
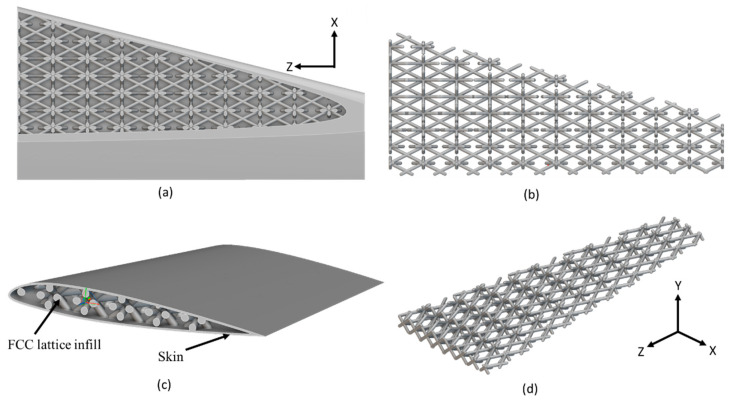
5 × 5 × 5 mm FCC lattice-infilled wing, (**a**,**b**) represents the plain view, (**c**,**d**) showing the isometric view with and without skin.

**Figure 9:**
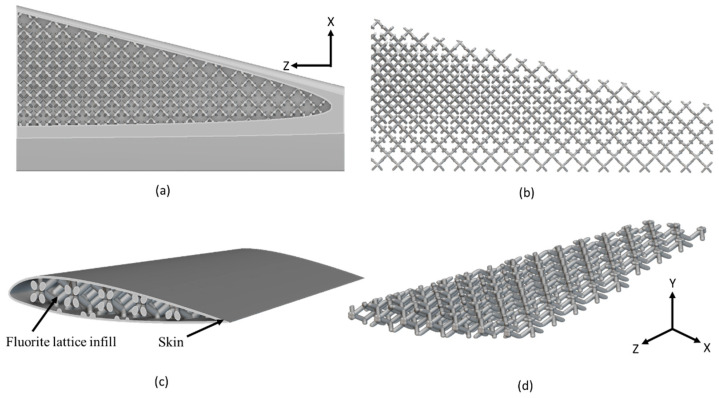
6 × 6 × 6 mm Fluorite lattice-infilled wing, (**a**,**b**) represents the plain view, (**c**,**d**) showing the isometric view with and without skin.

**Figure 10:**
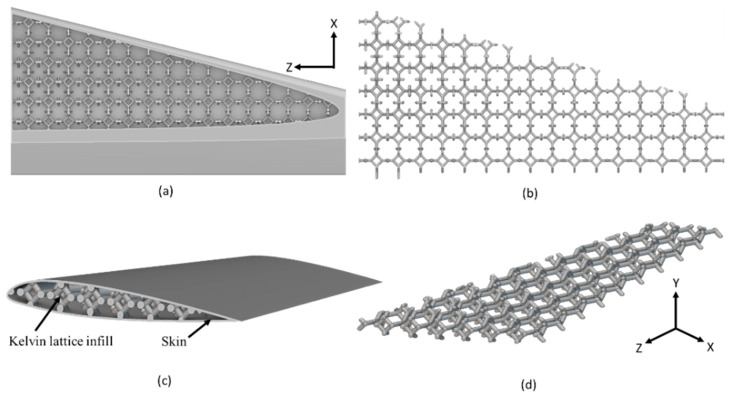
5 × 5 × 5 mm Kelvin infilled wing, (**a**,**b**) represents the plain view, (**c**,**d**) showing the isometric view with and without skin.

**Figure 11:**
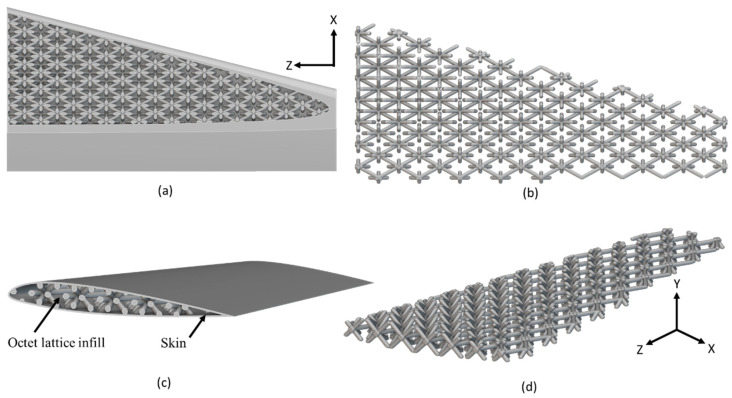
6 × 6 × 6 mm Octet lattice-infilled wing, (**a**,**b**) represents the plain view, (**c**,**d**) showing the isometric view with and without skin.

The final optimized arrangement of lattice-infilled wing structures for all types of lattices was obtained after an iterative process. Weight savings for each type were reported accordingly. The details of all these optimized wing designs are given in Figure 7, Figure 8, Figure 9, Figure 10 and Figure 11.

### 2.4. Numerical Simulation

**Figure 12:**
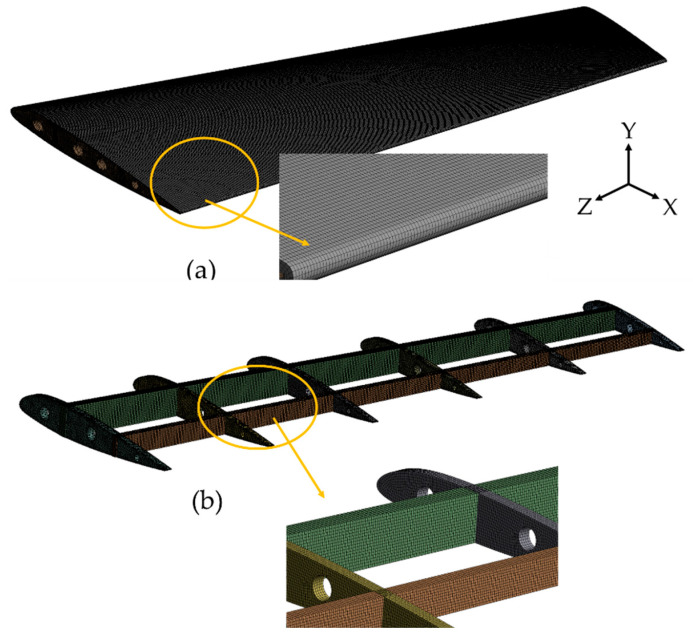
Finite element discretization of the wing structure (**a**) Skin (**b**) Internal structure.

**Figure 13:**
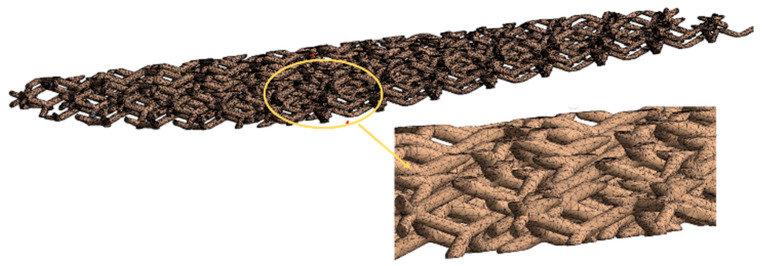
Finite element discretization of the lattice structure.

**Figure 14:**
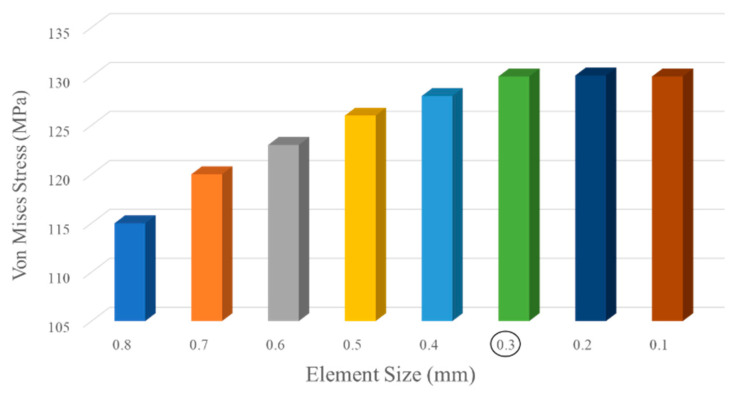
Mesh convergence study element size as function of maximum stress (von-mises).

**Figure 15:**
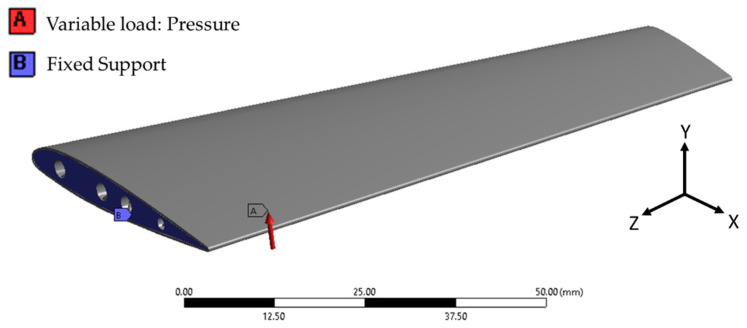
FE model boundary conditions fixed at the root and variable load applied along the axis (*z* axis) of the semi-wingspan.

The numerical simulation of the base conventional wing and optimized lattice-infilled wing structures were performed on the ANSYS workbench to investigate the elastic behaviors under design load. For the base-wing model, the whole structure is discretized with hexahedron mesh (Figure 12), while the lattice structures were discretized with second-order tetrahedral elements due to complex tiny structures (Figure 13). Considering the mesh convergence, a mesh size of 0.3 mm was set for wing structures, since from this element size the stress stabilizes at around 130 MPa, as shown in Figure 14. Decreasing elements less than 0.3 mm causes a significant increase in the computational time. The total number of mesh elements varied for each type of lattice, as shown in Table 3. The finite element simulations were performed on a dedicated workstation equipped with an AMD Ryzen 9 6800H with Radeon Graphics, 32 GB of RAM, and an NVIDIA GeForce RTX 3080 GPU. The simulations typically took between 1 and 2 h to complete, considering the complexity of the models and the desired level of detail in the results. The finite element model of the wing structure is shown in Figure 15. A fixed boundary condition was applied at the root of the wing, while the variable load, calculated (Figure 1) as per Schrenk’s approximation [54], is applied along the spar axis toward the tip side.

## 3. Results and Discussion

### 3.1. Lattice Optimum Configuration and Weight Saving 

**Figure 16:**
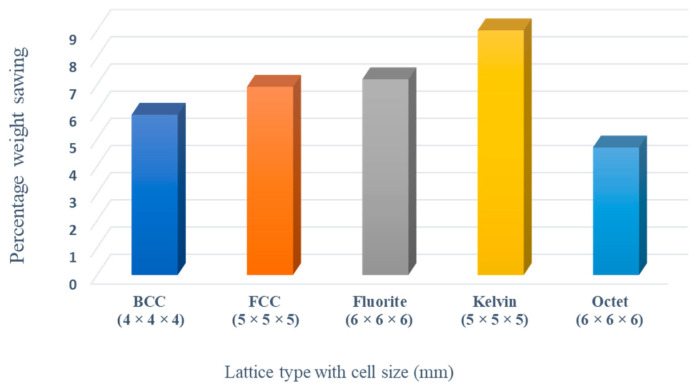
Percentage weight saving compared to the base-wing model.

The unit cell type and configuration study was performed for optimal lattice structure generation for the design load. Initially, the unit cell size arraignment of 10 × 10 × 10 mm was selected to be decreased until the design criteria were met (base-wing-tip deflection). Among different types of unit cells, as shown in Figure 3a of Section 2.3, five unit cells (Figure 4) named BCC, FCC, Fluorite, Kelvin, and Octet were selected based on weight saving and design response through an iterative process through a given optimization framework. A unit cell arrangement of 4 × 4 × 4 mm for the BCC unit cell type was obtained as an optimized lattice arrangement, followed by the 5 × 5 × 5 mm for FCC, 6 × 6 × 6 mm for Fluorite, 5 × 5 × 5 mm for Kelvin, and 6 × 6 × 6 mm for the Octet unit cell. The detailed arrangement and corresponding layouts are presented in Figure 7, Figure 8, Figure 9, Figure 10 and Figure 11, where the plain and isometric view of lattice-infilled optimized wing structures with and without skin are shown. It can be seen from these figures that at the maximum bending load, more material distribution is noted in the form of compact lattices at the area near the root of the wing, where the lattice structure is distributed as per the strength requirements in the form of branches, playing a structural role by making the wing stiffer [81]. The summary of weight saving compared to the base-wing model is presented in Figure 16. The maximum weight saving of around 9.5 percent was reported for the Kelvin cell type, while the lowest of 4.6 percent was for the Octet unit cell type. The FCC and Fluorite lattices reported about the same weight saving of around 7 percent, while the BBC saved up to 5.8 percent compared to the base-wing geometry. All the designs are considered lightweight compared to the base-wing model. These weight savings directly explain the significant enhancements in overall aircraft performance. The strategic placement of compact lattices near the wing root, supported by branches for structural reinforcement, enhances stiffness and durability. These weight savings contribute to improved aircraft maneuverability, fuel efficiency, and range capabilities—crucial factors for enhancing overall performance. Moreover, these lattice-infilled lightweight designs underscore their potential to positively impact maintenance and lifecycle costs, further enhancing the aircraft’s operational efficiency and sustainability. Furthermore, the utilization of lattice-infilled lightweight wing structures offers increased stiffness compared to traditional wing structures, further enhancing the overall performance of the aircraft.

### 3.2. Maximum Wing-Tip Deflection

**Figure 17:**
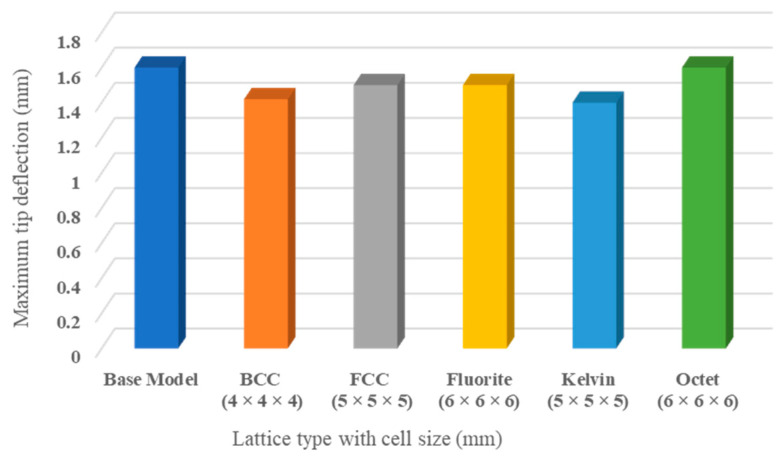
Comparison of maximum deflection for base-wing model and lattice-infilled wings.

The wing-tip deflection of lattice-infilled structures compared to the conventional wing design is presented here. The simulations were performed to measure the maximum deflection in the wing tip. Upon comparison, it was reported that the optimized wing lattice-infilled structures presented lower results than the base-wing model (1.6 mm). The maximum deflection of 1.5 mm was reported for FCC and Fluorite lattice structures, with a minimum of 1.42 mm for BCC and 1.4 mm for Kelvin lattice configurations. The Octet lattice structure produced almost the same deflection (of around 1.56 mm) as in the base-wing model. The less deflection in the BCC lattice is considered to be due to the close packing for the small cell configuration of 4 × 4 × 4 mm compared to other configurations. On the other hand, the close arrangement of beams in the Kelvin lattice unit cell makes it stiffer to deform. In the case of FCC, Fluorite, and Octet, the deflection is nearly equal to the base-wing deflection. Figure 17 summarizes the deflection reported at the wing tip in the loading direction. All the designs are considered safe under the design loading conditions as the maximum deflection is very small compared to the allowable deflection at extreme loading conditions (10% of half wingspan). This analysis of deflection in lattice-infilled wing structures offers crucial insights into their mechanical behavior, which directly impacts the overall structural integrity and safety of the aircraft. By comparing the deflection of optimized lattice structures to conventional wing designs, it is evident that the lattice-infused structures exhibit lower deflection values, indicating increased stiffness and resistance to deformation. Specifically, configurations such as the BCC and Kelvin lattice structures demonstrate minimal deflection, highlighting their potential to enhance structural robustness and safety margins. These findings are critical for ensuring the overall safety of the aircraft, as reduced deflection implies a lower strain on critical components during flight, thereby minimizing the risk of structural failure. Overall, the analysis indicates that the optimized lattice structures exhibit favorable mechanical properties, significantly enhancing the aircraft’s overall structural integrity.

**Figure 18:**
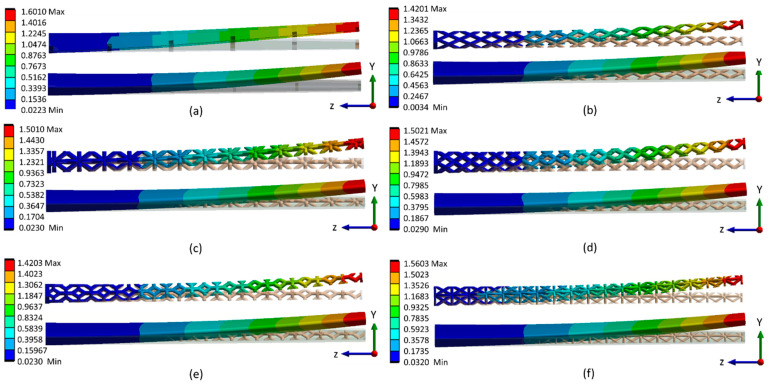
Comparison of maximum deflection (mm) in wing at different configurations (**a**) Base-wing model, (**b**) BCC lattice infill, (**c**) FCC lattice infill, (**d**) Fluorite lattice infill, (**e**) Kelvin lattice infill, (**f**) Octet lattice infill.

**Figure 19:**
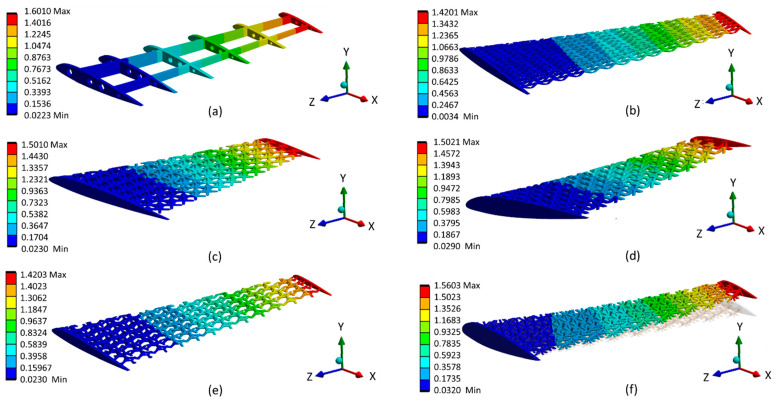
Isometric view of maximum deflection (mm) at wing tip a different configuration (**a**) Base-wing model, (**b**) BCC lattice infill, (**c**) FCC lattice infill, (**d**) Fluorite lattice infill, (**e**) Kelvin lattice infill, (**f**) Octet lattice infill.

Furthermore, linear elastic analyses of wing structures are presented in Figure 18 and Figure 19, where the deflection behavior of lattice-infilled wing structures is shown. As can be observed, the wing-tip deflection changes based on changing unit cell type and cell size. The comparatively less deflection in the case of the BCC (Figure 18b) and Kelvin (Figure 18e) lattice structures can be observed compared to the rest of the lattice structures. This establishes the dependency of tip deflection upon the lattice type and its arrangement in the design space.

### 3.3. Maximum Stress (von Mises) Distribution 

**Figure 20:**
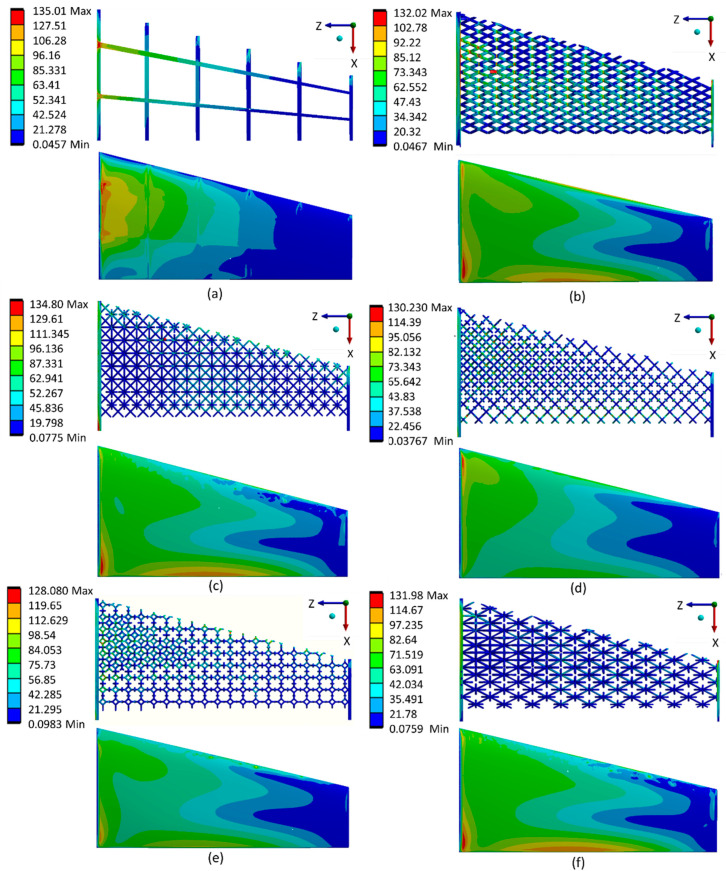
Stress distribution for (**a**) Base-wing model, (**b**) BCC lattice infill, (**c**) FCC lattice infill, (**d**) Fluorite lattice infill, (**e**) Kelvin lattice infill and (**f**) Octet lattice infill.

**Figure 21:**
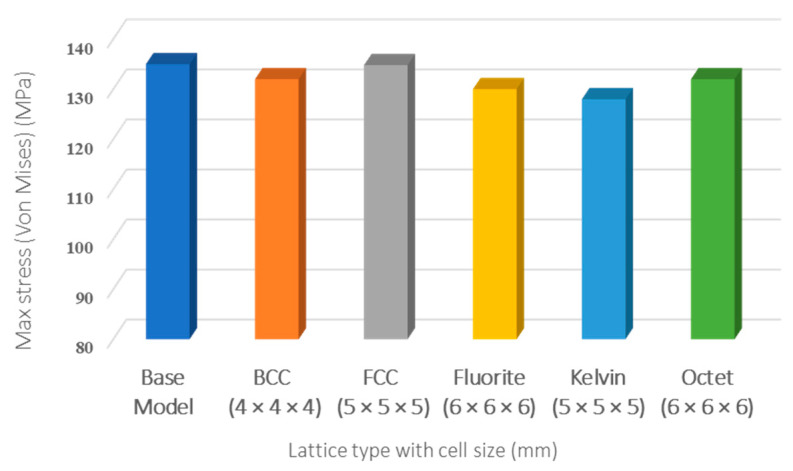
Summary of stress (von Mises) for different wing designs.

Investigations of the stress distribution and concentration among different types of optimized lattice-infilled wing structures were performed through linear elastic FEA on the ANSYS workbench. Figure 20 shows the stress distribution of each lattice structure and highlights that the stress distribution is dependent on the unit cell topology and its arrangement in the design space. It can be observed that the stress is uniformly distributed in the infilled lattice compared to the base-wing model, where the stress peak occurred in the spar end area (Figure 20a). This is due to the fact that in the lattice structure, the stress is carried by the distributed unit cells before passing into the entire structure. Among all lattices, the behavior of Fluorite, Kelvin, BCC, and Octet infilled lattice structures is better compared to the rest of the lattices. A lower maximum stress value of 128 MPa was reported for Kelvin followed by Fluorite with a maximum value of around 130 MPa. A higher stress value of around 134.8 MPa was recorded for FCC closely equals the peak stress of the base wing. The stress value for BCC and Octet lattice structure was around 132 MPa. A comparison of stress distribution over the skin can also be made from Figure 20. There is almost a similar distribution over the wing structure skin, with more stresses at the wing box area near the root, lowering towards the tip of the wing. BCC, FCC, and Octet infilled-structure skins were observed with a peak of stresses, as can be seen in Figure 20b,c,f. These stress peaks are presumed to be due to the open beam tips connected to the skin from the inside, causing stress concentration at the joint between the skin and the lattice beam. Furthermore, Figure 21 summarizes the peak values of stress measured for different types of lattice-infilled structures. All the stress values are within the design stress < 167 MPa (FoS 1.5), and the wing designs are considered safer. The relatively poor behavior of FCC may be due to the unit cell topology, where all the beams are connected to a single point on the face of the unit cell called a node, creating more stress concentration in the FCC unit cell type. More explanation of stress concentration is provided in the next section. 

### 3.4. Stress Concentration

**Figure 22:**
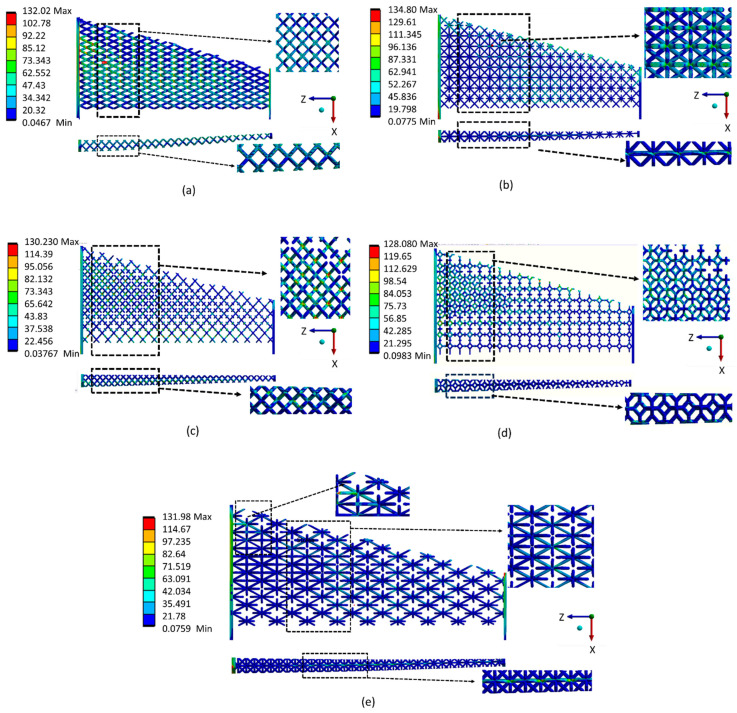
Stress concentration at different locations of optimized lattice-infilled structures, (**a**) BCC lattice infill, (**b**) FCC lattice infill, (**c**) Fluorite lattice infill, (**d**) Kelvin lattice infill and (**e**) Octet lattice infill.

The stress concentration in lattice structures as a result of elastic FEA is shown in Figure 22. The stress concentration area is near the root of the base wing, which is a very common phenomenon for cantilevers. In the case of lattice structures, the stress concentration is observed in the nodes and beams of the lattices. In almost all cases, the stress distribution is similar: higher at the root area with relatively uniform distribution over the whole wing structure. In the case of the Kelvin lattice, the stress distribution is seen in the beams of the lattice (Figure 22d). This uniform distribution of stress in the Kelvin lattice structure makes it a relatively better structure, where fewer stress peaks can be seen, due to a smaller number of open beams, compared to the other studied lattices in this study. In the case of FCC lattice stress, concentration is reported at the node point in the center of the unit cell where all the beams of the lattice are merging making it a more loaded area as can be seen in a closer image highlighted by dashed lines the Figure 22b. On the other hand, due to the number of beams in the BCC (Figure 22a), Fluorite (Figure 22c), and Octet (Figure 22e) lattice structures, the stress concentration is observed as a mix of nodes and beams. This distribution leads to a considerably stiffer behavior of the structures. This comparative study reveals that stress distribution and concentration are dependent on unit cell topology, its arrangement, and distribution over the design area, making it very interesting and important to consider in the design process while designing structures with lattices. This discussion on stress distribution emphasizes the critical role of lightweight construction and efficiency in enhancing the structural integrity and safety of aircraft. Notably, in lattice-infilled structures, stress is uniformly distributed compared to conventional wing models, where stress peaks typically occur in the spar end area. This uniform distribution indicates that lattice structures effectively distribute stress across distributed unit cells, thereby contributing to enhanced structural integrity. Additionally, the comparison of peak stress values for different lattice-infilled structures reveals that all stress values are within the design stress limits, indicating the safety of the wing designs. Overall, the stress distribution among lattice-infilled structures is generally favorable for structural integrity and safety, particularly during extreme loading conditions, providing a comprehensive understanding of the aircraft’s overall performance and safety profile.

## 4. Conclusions

This study performed a comparative analysis of different wing structures through a numerical approach. The structural behavior of different types of lattice-infilled wings with different unit cell types and arrangements was investigated, which reveals the feasibility of considering the replacement of the conventional spar–ribs wing structure design with non-conventional infilled lattices. In this study, five different types of unit cells were used for the lattice infill of wing structures. An iterative design process through automatic Python coding using commercial design software (nTop and ANSYS workbench) was performed to obtain the optimized infilled structures through the design variable of the unit cell type and its arrangement in the design space. A significant weight reduction in lattice-infilled designs was reported compared to the conventional base-wing design. The highest weight saving of 9.5% for the Kelvin structure was reported among all lattice structures compared to the conventional wing. The optimized lattice-infilled structures were further analyzed for wing-tip deflection and stress distribution. It was revealed that the maximum deflection of all infilled-lattice wing designs was under the design value. The lowest minimum deflection of 1.4 mm was reported for the Kelvin lattice-infilled structure. Similarly, a stress distribution study was performed, where an almost similar distribution of stress over the skin was reported for all designs, while within the lattice structures, a different distribution over nodes and beams was observed. Dependency of the stress concentration over the unit cell topology and its arrangement was established. A reduction of 7 MPa in the max stress (von Mises) was observed for the Kelvin lattice, and this was up to 5 MPa for the rest of the lattice structures, except for FCC, where a similar stress value was recorded compared to conventional base-wing design. Among all types of lattice structures, the Kelvin is considered the feasible choice for current applications, with good weight saving and lower deflection and stress values. Considering the better performance (lower stress and deflection values) of the rest of the lattices, infills can also be considered for the complete replacement of the conventional spar–rib wing structure. However, experimental validation is required for further design exploration of the structures for an absolute optimal design. Furthermore, as a limitation of this study, the effect of lattice beam thickness and orientation also needs to be considered in future studies. Overall, this study demonstrates the feasibility of replacing conventional spar–ribs wing structures with lattice-infilled structures, offering lightweight solutions and enhanced performance. By prioritizing weight savings and stress distribution, we contribute to greener aviation, improving efficiency and sustainability goals with lighter aircraft designs. Thanks to additive manufacturing, this lattice structure fabrication is possible with less time and benefits from easy prototyping and more design complexity and freedom. The making of single-wing structures without making assemblies has always been a point of interest in the industry, including at Airbus with the project ”Wing for tomorrow” [82]. The applications of this study can be extended further to other parts of aircraft such as winglets and horizontal and vertical tails.

## Figures and Tables

**Table 1:** Important parameters for the UAV wing.

Specification	Value
Wingspan (b) [mm]	200
Chord Length (Cr) Root [mm]	50
Chord Length (Ct) Tip [mm]	25
Area of the Wing (s) [m^2^]	0.0075
Aspect Ratio (A)	5.3
Taper Ratio (λ)	0.5
Maximum Weight of the Fixed Wing UAV [kg]	5
Maximum Load Factor (n)	5.7
Cruise Altitude (H) [m]	120

**Table 2:** Mechanical properties of AlSi10Mg [74].

**Property**	**Value**
Young’s modulus [MPa]	68,000
Density [kg/m^3^]	2650
Poisson’s ratio	0.33
Tensile strength (Yield) [MPa]	251

**Table 3:** Finite element discretization detail of different types of lattices.

	BCC	FCC	Fluorite	Kelvin	Octet
Total No. of Nodes	791,639	282,432	791,639	1,131,328	6,321,189
Total No. of elements	337,513	426,279	337,513	495,880	4,017,422

## Data Availability

Data are contained within the article.
